# Bladder Cancer Immunotherapy in 2025: ALBAN, CREST, POTOMAC, and the Winner Is BCG!

**DOI:** 10.1590/S1677-5538.IBJU.2025.9921

**Published:** 2025-12-20

**Authors:** Leonardo O. Reis

**Affiliations:** 1 Universidade Estadual de Campinas Faculdade de Ciências Médicas UroScience Campinas SP Brasil UroScience, Faculdade de Ciências Médicas, Universidade Estadual de Campinas (UNICAMP), Campinas, SP, Brasil; 2 Pontifícia Universidade Católica de Campinas ImunOncologia Campinas SP Brasil ImunOncologia, Pontifícia Universidade Católica de Campinas (PUC-Campinas), Campinas, SP, Brasil; 3 Tecnologia e Inovação em Câncer Geniturinário Instituto Nacional de Ciência INCT UroGen Campinas SP Brasil INCT UroGen, Instituto Nacional de Ciência, Tecnologia e Inovação em Câncer Geniturinário, Campinas, SP, Brasil

## COMMENT

Immune checkpoint inhibition (ICI) has transformed treatment paradigms in metastatic and muscle-invasive bladder cancer over the past decade. The integration of ICIs into earlier stages of urothelial cancer has long been viewed as an inevitable evolution in bladder cancer therapeutics. In 2025, this assumption faces its first major test in BCG-naïve high-risk non-muscle-invasive bladder cancer (HR-NMIBC) with the publication of the POTOMAC and CREST trials. These Phase III studies, which are large, rigorously conducted, and use induction plus 2 years maintenance BCG in their control arms, allow a critical reassessment of whether systemic (1 year) or subcutaneous (2 years) checkpoint blockade meaningfully enhances outcomes beyond BCG monotherapy (1, 2).

While POTOMAC and CREST reached statistical significance for their primary endpoints, the absolute magnitude of benefit demands careful interpretation. Both studies met their primary endpoints, showing improvements in disease-free or event-free survival. Yet, when placed in a clinical context, these results challenge rather than confirm the expectation that systemic or injectable checkpoint inhibition should be added to frontline BCG for all patients. Here, we critically examine what these trials have taught us scientifically, clinically, and pragmatically, and why BCG remains the backbone of NMIBC immunotherapy despite the biological elegance of checkpoint inhibition.

The results are clear and consistent: while the addition of ICIs produces a statistically significant improvement in disease-free survival (DFS) or event-free survival (EFS), the absolute benefit is modest, toxicity increases substantially, and there is no clue for improvement in the endpoints that matter most, progression, metastasis, bladder preservation, and survival.

Considering therapy-related adverse effects (TRAE) grade ≥3 at 36 months, and EFS/DFS, the number needed to harm (NNH) was 4 and 6, and the number needed to treat (NNT) was 23 and 14, for Sasanlimab and Durvalumab, respectively. These endpoints represent the outcomes of the highest value to patients and health systems, raising the critical question of clinical meaningfulness, particularly when the marginal gains come at the cost of significant toxicity and financial burden (3).

MIBC and/or metastatic disease was 4.7% in durvalumad + BCG vs. 4.4% in BCG alone and 2.8% in sasanlimab + BCG vs. 3.9% in BCG alone. Moreover, historically, most NMIBC-related deaths arise from progression to muscle-invasive disease. The failure of checkpoint intensification to influence this trajectory should temper enthusiasm for broad adoption and refocus the field toward precision immuno-oncology rather than universal escalation.

Immune-related adverse events, including hepatotoxicity, endocrine dysfunction, and gastrointestinal or pulmonary inflammation, often require corticosteroids, specialist care, and sometimes lead to irreversible morbidity. Additionally, ICI therapy imposes a significant economic burden due to drug acquisition costs, infusion or administration infrastructure, monitoring and immunotoxicity management, as well as increased imaging and laboratory surveillance.

Most importantly, POTOMAC and CREST reaffirm the foundation upon which NMIBC care has rested for decades: BCG alone, when administered with induction and maintenance, remains a potent and reliable immunotherapy. The 36- and 24-month EFS/DFS rates in the BCG-only arm, with full induction and maintenance, were 74.8% and 79.9% in CREST, and 77.4% and 81.6% in POTOMAC, respectively. Additionally, the optimized conditions in contemporary clinical trials resulted in a higher-than-expected complete response rate at any time and unusually low grade 3-4 adverse events for the BCG alone group, with rates of 93% and 4% in the POTOMAC trial and 85.2% and 6% in the CREST trial, respectively, significantly superior to the historical series (1, 2, 4).

Given the safety and strong performance of BCG alone, the absolute gain on disease/event free survival in 36 and 24 months was 4.4 and 4.9% in POTOMAC (durvalumab 1y 13cycles + BCG, with 17% absolute increase in Grade ≥3 TRAE) and 7.3 and 4.8% in CREST (sasanlimab 2y 25cycles + BCG, with 23% absolute increase in Grade ≥3 TRAE), respectively (1, 2).

Importantly, a third arm (ICI + BCG induction only) in both the CREST and POTOMAC trials failed to outperform the complete 2-year BCG induction-plus-maintenance schedule, confirming that BCG monotherapy, when adequately offered, maintains an exceptional therapeutic index, high efficacy, and minimal severe toxicity, setting a high bar for any combination strategy. This finding aligns with the randomized phase III ALBAN trial, which offered 1 year of BCG maintenance and did not demonstrate an improvement in EFS compared to BCG alone in BCG-naive high-risk NMIBC patients (1, 2, 5).

While endpoint definitions and censoring rules for EFS (e.g., upper tract tumors, low-grade recurrences) vary across studies, limiting direct comparisons, other important factors, such as patient mix, geographic distribution, and BCG strain availability, may also influence the results. While the POTOMAC and CREST trials (1, 2) recruited patients with significant previous BCG vaccine and tuberculosis exposures (i.e., Russia), the ALBAN trial (5) limited its recruitment to areas with no or minimal previous BCG/tuberculosis exposure (France, Belgium, and Spain) (6).

In the future, we may need to optimize patient selection based on biomarkers that predict their immune responses and on both BCG-related and unrelated immunogenicity, making the BCG naïve definition more comprehensive, considering any previous BCG priming beyond intravesical BCG. A major challenge is predicting the immunogenic potential of immunotherapies (BCG and beyond) and how it varies with environmental factors (high vs. low-income countries), comorbidities, and age, as it influences not only treatment response but also toxicity (7). For better results, we may evolve into a system of "immune staging" for a more individualized approach, complementing the current tumoricentric and immune-blind approach.

Even patients treated with BCG alone conceal inherent heterogeneities. Based on current knowledge, realistic strategies to potentially enhance the already robust BCG alone results include higher rates of treatment completion and the extension of BCG maintenance for up to 3 years (8, 9). Even in the CREST and POTOMAC trials, BCG treatment completion rates were limited to 53% to 54%. Additionally, 65 to 78% of patients received BCG strains other than TICE, each containing a wide variation of 1 to 8 × 10^8^ colony-forming units (1, 2).

In 2025 trials (1, 2, 5) also highlight a significant limitation in bladder cancer immunotherapy research: the lack of reliable predictive biomarkers. PD-L1 expression remains insufficient to guide treatment selection. No validated genomic signature or immune profiling strategy emerged. Translational correlatives released so far do not clarify who benefits most from systemic therapy. Without meaningful biomarkers, systemic immunotherapy in NMIBC becomes an unguided escalation strategy, exposing many patients to toxicity without assured benefit.

The ongoing KEYNOTE-676 trial will present results of BCG induction, followed by 3 years of maintenance, both with and without intravenous pembrolizumab (administered for 2 years), in patients with recurrent or persistent high-risk NMIBC (9). Future trials on immunotherapy must be robustly designed with strategies for identifying biomarkers and understanding immunological profiles ("immuno-score") that predict clinically significant outcomes (bladder preservation, progression, metastasis, survival), and serious side effects.

### Future clinical and research implications

BCG monotherapy should remain the standard of care for BCG-naïve HR-NMIBC. Checkpoint inhibitors should not be universally integrated into first-line NMIBC treatment. Patient selection must be prioritized, with shared decision-making that transparently weighs modest benefits against toxicity and cost. Future research must be biomarker-driven, incorporating immune profiling, genomic classifiers, and spatial immunology. Mechanistic studies should elucidate why BCG remains so effective and to early detect which patients truly require treatment beyond BCG. Alternative immuno-oncologic strategies, such as intravesical cytokine engineering, oncolytic vectors, or localized immune modulation, may offer superior therapeutic ratios compared with systemic ICIs. Only through such approaches can we deliver meaningful advances in bladder preservation, progression prevention, and survival.

**Figure 1:**
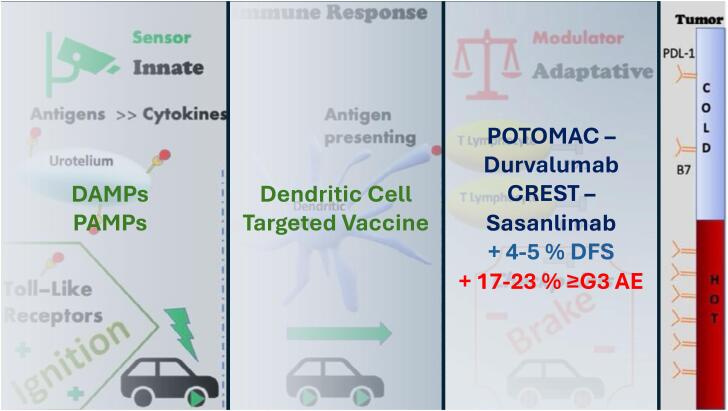
Co-dependency diagram of innate ("ignition", TLR) and adaptive ("brake", checkpoint) immune responses as a multi-targeted immunotherapy strategy (developed by the author).

DAMPs = Damage-Associated Molecular Patterns; PAMPs = Pathogen-Associated Molecular Patterns; DFS = disease-free survival (increment); G3 AE = grade 3 adverse effect (increment)

## CONCLUSIONS

One of the most important insights from both positive trials is the high efficacy of contemporary BCG when delivered with full induction and maintenance, consistent with international guideline standards. Checkpoint inhibitors are expensive and potentially toxic; deploying them empirically, without biomarker guidance, is no longer acceptable in modern immuno-oncology. Precision strategies, immune profiling, molecular subtyping, spatial immunophenotyping, and circulating biomarkers must become central in future NMIBC trials.

In high-income settings, these costs strain oncology budgets. In middle-income countries, including Brazil, where bladder cancer incidence is rising, and access to immunotherapy remains uneven, such regimens risk exacerbating disparities (10). Without clear evidence of improved progression or survival, the cost-effectiveness of adding ICIs to BCG is questionable. Health-policy considerations, particularly in universal health systems, require that incremental benefits justify broad public investment.

BCG should remain the foundation of care, and systemic ICIs should be used sparingly, thoughtfully, and, ideally, guided by biomarkers that do not yet exist. The field must now pivot from empiric combination toward precision immunology and rational patient selection.

The "winner" is therefore not a single drug, but the strategy that centers on BCG as the necessary immunologic partner, unlocking an era of bladder-sparing precision immunotherapy. This approach will include a more individualized treatment, where, beyond tumor characterization, understanding the patient's "immune staging" will play a crucial role for treatment efficacy and safety.

## Data Availability

Uninformed
